# Comparison of inter- and intraspecies variation in humans and fruit flies

**DOI:** 10.1016/j.gdata.2014.11.010

**Published:** 2014-11-22

**Authors:** Juliann Shih, Russ Hodge, Miguel A. Andrade-Navarro

**Affiliations:** a Department of Biology, Massachusetts Institute of Technology, Cambridge, MA, USA; b Broad Institute of Harvard and Massachusetts Institute of Technology, Cambridge, MA, USA; c Max Delbrück Center for Molecular Medicine, Germany; d Faculty of Biology, Johannes-Gutenberg University of Mainz, Mainz, Germany; e Institute of Molecular Biology, Mainz, Germany

**Keywords:** Evolution, Population, Variation, Human genome, *Drosophila*

## Abstract

Variation is essential to species survival and adaptation during evolution. This variation is conferred by the imperfection of biochemical processes, such as mutations and alterations in DNA sequences, and can also be seen within genomes through processes such as the generation of antibodies. Recent sequencing projects have produced multiple versions of the genomes of humans and fruit flies (*Drosophila melanogaster*). These give us a chance to study how individual gene sequences vary within and between species. Here we arranged human and fly genes in orthologous pairs and compared such within-species variability with their degree of conservation between flies and humans. We observed that a significant number of proteins associated with mRNA translation are highly conserved between species and yet are highly variable within each species. The fact that we observe this in two species whose lineages separated more than 700 million years ago suggests that this is the result of a very ancient process. We hypothesize that this effect might be attributed to a positive selection for variability of virus-interacting proteins that confers a general resistance to viral hijacking of the mRNA translation machinery within populations. Our analysis points to this and to other processes resulting in positive selection for gene variation.

## Introduction

Traditionally, we have traced the evolution of genes by comparing homologous versions in different organisms. Such homologies reflect a basic conflict: between the conservation of sequence features related to gene functions and to the structures of translated protein products on the one hand, and on the other, processes that produce genetic variation and make sequences drift away over millions of years of evolution.

With the sequencing of multiple versions of genomes of single species, we have now the chance to observe a different aspect of the forces that shape molecular evolution by studying gene sequence variation within a species. There is a general expectation that the variation of a gene's sequence *within* a species and *between* species will agree, leading to a similar constraint of evolutionary drift at both levels. However, we wondered whether we could detect particular genes that displayed increased variability within a single species, for example to provide fast adaptation of a population to variable environments, or to escape pathogens that recognize a protein and co-evolve with the species. Such genes might be detected by rates of (evolutionary short-range) intraspecies variability that are higher than expected when compared to (evolutionary long-range) interspecies variability.

To explore whether such cases can be detected through an unbiased, genome-wide level analysis, we took advantage of the recent evaluation of genetic variation in human [1] and *D. melanogaster* [2] genomes. To contrast short-range intraspecies variation with interspecies variation, we obtained and concentrated our analysis on pairs of one-to-one orthologous genes between these two species. Then we defined the degree to which each pair of human and fly orthologs demonstrated identity to each other and their respective intraspecies variation. Comparisons of the results allowed us to find, overall, the expected correlation between long and short evolutionary range conservation: most genes are highly constrained in their evolution and therefore will not change much both within and across species. However, we could also detect outlier genes that are highly variable in human or fly populations while being highly conserved between these species. We carried out the analysis both at the levels of nucleotide sequences and at the (translated) amino acids to compare and distinguish evolutionary constraints that might possibly act differently on these levels.

## Results

The evolutionary divergence of *Homo sapiens* and *D. melanogaster* (fruit-fly) from a common ancestor has been estimated at approximately 782.7 million years ago [3]. Despite this, Ensembl Compara's phylogenetic-approach homolog prediction tool [4] indicates that a total of 14.9% of human genes and 46.0% of fly genes have orthologs (genes in different species that descended from the same ancestral sequence) to one or more fly and human genes, respectively.

A significant challenge in evolutionary biology is to determine the relationship between a gene's functions and its ability to avoid being phased out on an evolutionary scale, due to either negative selection or, more likely, disuse. Also implicit in such relationships is the level of conservation between human genes and their fly ortholog(s) and vice versa, which can be calculated in terms of the percentage of identity of nucleotides and/or amino acids. According to current thinking, conservation between a fly and human ortholog pair would imply that both genes have a function implicated in the survival of each species, while mutations potentially result in phenotypic disadvantages. Although we are aware of no studies that have proven this in fruit-flies, a high correlation has been established between the essential functions of mouse genes and their level of evolutionary conservation in humans [5]. Thus between different human individuals, as well as between different fly strains, high interspecies DNA/amino acid transcript conservation should indicate “essential” gene functions and should also confer high intraspecies conservation in the same gene.

The advent of next-generation sequencing technologies has made the full sequencing of the genomes of large numbers of individuals and strains significantly more efficient. Two large-scale projects taking advantage of these technologies have been Bart Deplancke's catalog of insertions, deletions, complex variants, and single nucleotide polymorphisms (SNPs) in 39 *D. melanogaster* Genetic Reference Panel (DGRP) inbred lines and their effects on gene expression [2], and the 1000 Genomes Project, which catalogs variants from 1092 human individuals from 14 different populations [1]. The 1000 Genomes Project also shows that evolutionary conservation is a key determinant of the strength of purifying selection, meaning that there is a correlation between the essential nature of the functions of a protein-coding gene and the conservation of base pairs (or corresponding amino acids) that it exhibits among different individuals of the same species.

Because evolutionary essentiality has been shown to cause both intra- and interspecies conservation (and therefore to restrict variation), here we strive to formally establish a correlation between percentage identity between human–fly orthologs (taken from reference genomes GRCh37.p12 and BDGP5) and intraspecies variation (taken from the DGRP and 1000 Genomes Project), while developing an analysis that would point to any genes that might escape this “rule”.

**Fig. 1:**
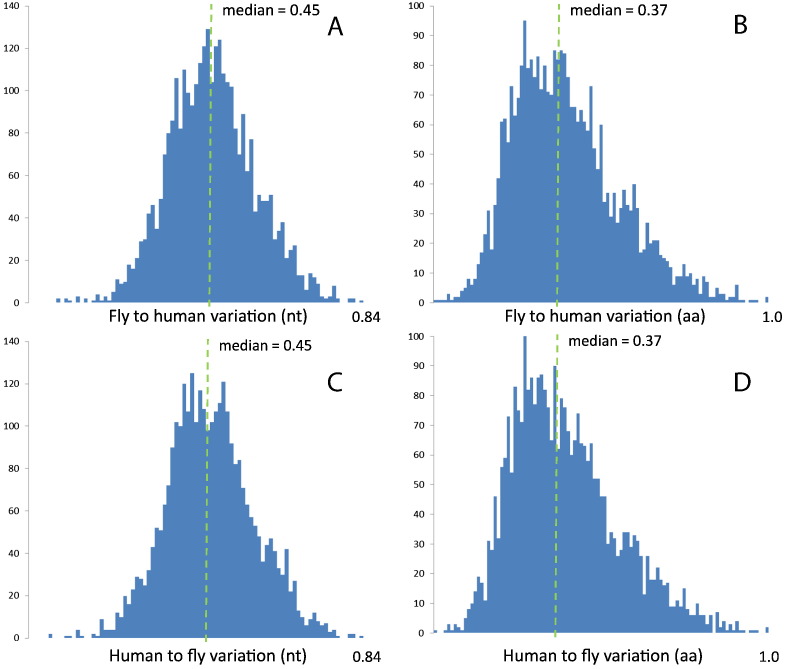
Distributions of interspecies similarity (in percentage identity). Fly to human in nucleotides (A) and amino acids (B). Human to fly in nucleotides (C) and amino acids (D).

To accomplish this, we determined the intraspecies variation and interspecies percentage identity of 3082 one-to-one orthologous pairs of genes between *H. sapiens* and *D. melanogaster* (Supplementary Table 1; see Methods for details). For each of the orthologous pairs, fly-to-human and human-to-fly percentage identities (for both nucleotides and amino acid sequences) were determined (Fig. 1; see Methods for details). For nucleotides, the distributions are rather symmetrical and show a peak at around 45% identity, while for amino acids the peak percentage identity is slightly lower, at around 30%, and the distributions show a skew to the left (towards lower values of identity).

**Fig. 2:**
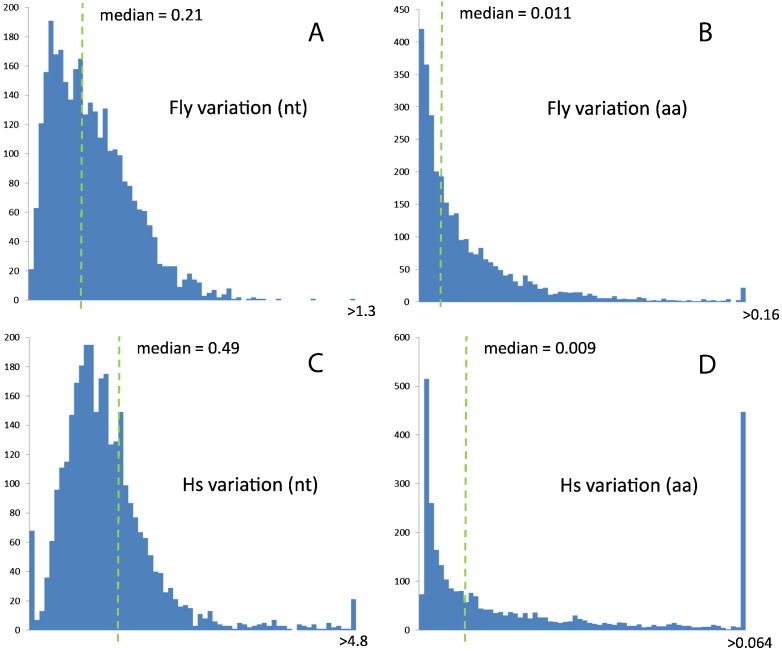
Distributions of intraspecies variation values. Fly genes in nucleotides (A) and amino acids (B). Human genes in nucleotides (C) and amino acids (D).

We also computed the intraspecies variation score calculated for each gene, normalized for the length of the gene (Fig. 2; see Methods for details). The nucleotide distributions present a maximum, whereas the amino acid distributions peak near zero variation. This difference is due to the nucleotide variability allowed by synonymous substitutions in protein-coding genes. The median for the nucleotide distribution is higher for humans than for flies, whereas the medians of the amino acid distributions are rather similar. Comparing distributions of intraspecies variation is problematic because of fundamental differences in the geographic distribution of the human and fly populations that were chosen (see Discussion).

We found a correlation of intra-species variation between orthologous genes; that is, if the human and the fly genes had high intraspecies variation the corresponding fly ortholog also had high intraspecies variation, as expected from the conservation of functional essentiality of ortholog genes across species. This correlation was weaker when considering conservation in nucleotides (Pearson's R = 0.0307, two-tailed p-value = 0.0880, m = 0.00646, b = 0.229) than in amino acids (Pearson's R = 0.0767, two-tailed p-value = 2.04e− 0.5, m = 0.0337, b = 0.0214) (data not shown). The higher correlation when comparing amino acid conservation could be expected from the stronger functional constraints applying to protein sequences when compared to their coding genes.

**Fig. 3:**
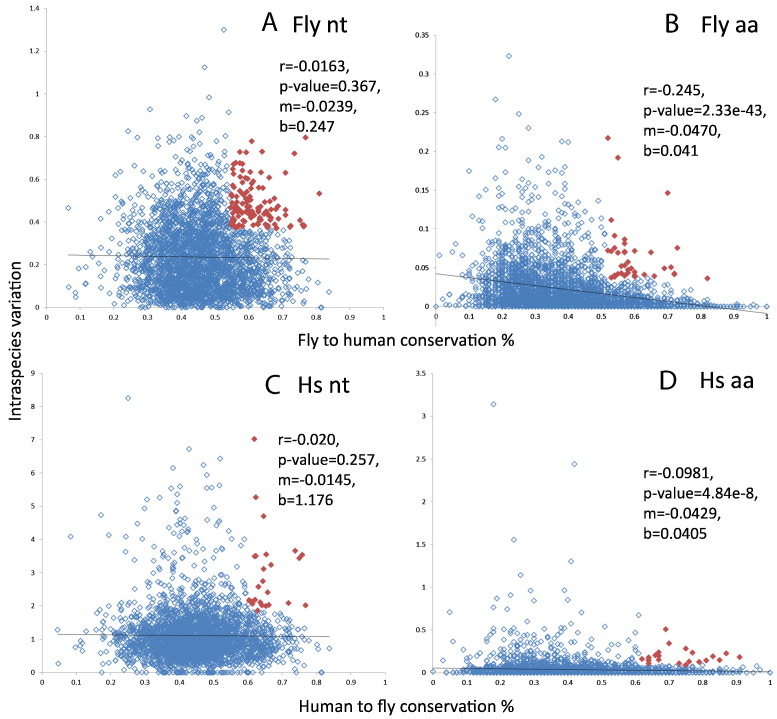
Distribution of intra- versus interspecies conservation. Cut-offs were applied to select sets of genes with high intraspecies variation and high interspecies conservation (filled diamonds). (A) Fly intra- versus interspecies (nt): cut-offs nt ≥ 0.36863452 and nt ≥ 0.544397227. (B) Fly intra- versus interspecies (aa): aa ≥ 0.036343094 and aa ≥ 0.49. (C) Human intra- versus interspecies (nt): nt ≥ 1.867292351 and nt ≥ 0.603982301. (D) Human intra- versus interspecies (aa): aa > = 0.0893 and aa ≥ 0.62. Regression lines (black) are shown for each distribution indicating r value for Pearson 's correlation, two tailed p-value, and the slope (m) and value at y = 0 (b) of the regression line.

Finally, we compared intraspecies variation versus interspecies percentage identity (Fig. 3). A negative correlation was found between these values. This negative correlation can be expected since essential genes can be expected to have lower intraspecies variation and higher interspecies conservation. Again, the observed correlations were stronger for amino acids than for nucleotide conservation.

Next, we focused our analysis on fly or human genes that, while exhibiting high conservation to the other species, displayed high values of intra-species variation. We show the distribution of such values and the cut-offs chosen for gene selection in Fig. 3. The cut-offs were chosen for human genes, so that genes were both among the top 10% most conserved among species and among the top 10% most variable within the human genomes; this resulted in 28 and 39 genes when considering nucleotides and amino acids, respectively. Applying a similar cut-off resulted in too few genes in the fly for further statistical analysis, so we used a 20% cut-off; this resulted in 118 and 39 genes when considering nucleotides and amino acids, respectively.

Gene ontology enrichment analysis of the selected genes using GOstat [6] indicated certain functions that were significantly enriched (using Benjamini correction, annotation database fb or goa_human, for fly and human genes, respectively, and using as background the set of 3082 fly or human genes used in the analysis; Table 1). These functions were mostly related to mRNA translation and protein synthesis: genes encoding constituents of the ribosome were found whether considering conservation in nucleotides or in amino acids, and aminoacyl tRNA synthetases were particularly found in the set of fly genes when amino acid conservation was considered.

Ribosomal proteins are highly conserved between humans, *Drosophila*, *Caenorhabditis elegans* and *Saccharomyces cerevisiae* [7]. However, here we find that some are highly variable within humans and *Drosophila*. This result could be explained by an “arms race” between eukaryotic organisms and viruses, in which eukaryotes generate variable versions of the proteins of the mRNA translation machinery, which needs to be hijacked by viruses in order to produce their own proteins [8], while keeping their general structural features highly conserved.

When comparing the lists of selected genes by nucleotide conservation in humans and flies, just eight gene pairs appear in both lists: GFM1/ico, OSGEP/CG4933, POLR2E/Rpb5, PRDX4/Jafrac2, QDPR/Dhpr, RPS9/RpS9, SRP54/Srp54k and TSTA3/Gmer. The overlap is even smaller when considering amino acid conservation: OSGEP/CG4933 again, and a new pair, GCAT/CG10361. This small amount of overlap in genes contrasts with the similar functional enrichment observed between the gene lists for flies and humans. We seem to be detecting the selection of variation in genes that perform given functions both in humans and flies, but these functions do not involve precisely the same proteins. This can be expected if we are dealing with a process triggered in response to viruses that have been evolving at least over 700 million years: it is predictable that they must modify their targets, but they cannot modify the overall need to target the mRNA translation machinery itself.

Regardless, several of the nine gene pairs mentioned above are related to mRNA translation. The human gene OSGEP encodes the O-sialoglycoprotein endopeptidase, whose fly ortholog, CG4933, does not seem to have been functionally validated through experimental evidence. The name of this protein is misleading as it is probably a tRNA N6-adenosine threonylcarbamoyltransferase  — that is, a protein involved in tRNA synthesis and is thus once again related to mRNA translation.

Ribosomes consist of a small 40S subunit and a large 60S subunit which include approximately 80 proteins. RPS9 encodes the ribosomal protein S9, in the 40S subunit. Its structure and position within the 40S subunit can be appreciated in the structure of the 40S subunit for *Drosophila* [9]: chain J in PDB record 3J3A; it interacts with the 18S ribosomal RNA and the 40S ribosomal proteins S15a, S24, S2, S30 and S4 (according to the corresponding NCBI's MMDB structure summary). It is located on the solvent side of the 40S.

GFM1 (a.k.a. EGF1) encodes one of the mitochondrial translation elongation factors. POL2RE (a.k.a. RPB5) encodes one of the 12 subunits of the RNA polymerase 2; this protein has an exposed domain that interacts with the Hepatitis B virus X (HBx) transcriptional regulator [10].

The remaining pairs of orthologs identified as outliers both in humans and flies (following the principle of intraspecies/interspecies similarity) do not seem to be directly related to mRNA translation. PRDX4 encodes periredoxin 4, whose ortholog in the fly is Jafrac2 thioredoxin peroxidase 2. PRDX4 probably has a function in facilitating protein folding by disulfide bond formation in the endoplasmic reticulum [11]. QDPR encodes the quinoid dihydropteridine reductase involved in the recycling of tetrahydrobiopterin (BH4), the cofactor of the aromatic amino acid hydroxylases [12]. SRP54 is part of the signal recognition particle, a ribonucleoprotein complex that targets secretory proteins to the ER and interacts with the nascent signal peptide during mRNA translation [13].

TSTA3 encodes a GDP-L-fucose synthase [14], 61% identical in sequence to the *Escherichia coli* ortholog, which is involved in the biosynthesis of GDP-fucose. Fucose is a member of the family of glycoconjugates, which include glycoproteins and glycolipids. The protein encoded by the orthologous gene in the fly, Gmer (a.k.a. CG3495) was identified in a *Drosophila* screen for genes involved in the uptake of exogenous dsRNA for gene silencing by RNA interference [15], a process that is conserved in *Drosophila* and *C. elegans*.

The ninth orthologous pair, which appears when considering amino acid conservation, is GCAT/CG10361. GCAT is the glycine C-acetyltransferase, whose product is a mitochondrial protein that catalyzes a step in the degradation of threonine to glycine.

Interestingly, amino acid metabolism is the most enriched GO term in the selected list of fly genes when considering amino acid conservation, with eight genes (p-value 2.32e− 07) and including the GCAT/CG10361 pair. It also includes five tRNA-synthetases and two other genes, GCDH/CG9547 and AMT/cg6415. AMT is the aminomethyltransferase, one of four critical components of the glycine cleavage system. GCDH is the glutaryl-CoA dehydrogenase, which participates in the catabolism of lysine, hydroxylysine, and tryptophan.

Considering that GO annotations can be improved using associations between databases [16], we computed the enrichment of fly genes using the annotation of the corresponding human orthologs. For the GO term amino acid metabolism, this resulted in the selection of ten genes and a better p-value (GOstat, goa_human annotations, Benjamini corrected, p-value 1.46e − 08, 10 genes of 474: GARS, NARS, RARS, ASL, GCAT, QARS, MCCC2, EPRS, SGSH, FAH). Three gene pairs were newly selected: FAH/faa, MCCC2/CG3267 and ASL/CG9510.

FAH is the fumarylacetoacetate hydrolase, the last enzyme of the tyrosine catabolic pathway. ASL is the argininosuccinate lyase, which catalyzes the reversible hydrolytic cleavage of argininosuccinate into arginine and fumarate. MCCC2 is the methylcrotonoyl-CoA carboxylase 2 (beta), involved in leucine catabolism.

At this point, we cannot assert whether the observed enrichment in genes with a function in amino acid metabolism has a biological meaning in the context of our analysis, or whether this effect occurred through the concurrent selection of several tRNA synthetases and enzymes of amino acid catabolism, both annotated as related to amino acid metabolism.

If this observation is biologically relevant, it means that the *Drosophila* population whose members were sequenced is under positive selection for an increased variability in gene functions related to amino acid catabolism and tRNA synthesis. While we can imagine that the effect on tRNA synthesis could be related to viral interactions, we do not see such a connection in the case of amino acid catabolism. Instead, another explanation could be a divergence that permits the survival of the population as it experiences rapid variations in environmental sources of amino acids.

## Discussion

We have produced a resource that points to genes that have been highly conserved over long evolutionary distances but which exhibit high variation within flies or humans. We did this by isolating a total of 3082 one-to-one orthologous fly and human genes and analyzing their percentage identity by comparing reference genomes GRCh37.p12 and BDGP5, and their intraspecies variation.

Highly conserved genes are enriched in functions essential for the organism. We observed that if from those highly conserved genes, we select those that are most variable within a species, some of those essential functions remain significant, namely mRNA translation-related functions, when considering fly or human genes, when considering either nucleotide or amino acid conservation.

The results we reported reflect enrichment based on relatively strict cut-offs that resulted in small selections of genes, using the genes that are both among the top-most conserved between species and among the top-most variable within species (top 10% and top 20% for humans and flies, respectively). At a less restrictive cut-off of 50%, which selected 772 and 580 genes for flies (considering nucleotide or amino acid conservation, respectively) or 766 and 746 genes for humans (considering nucleotide or amino acid conservation, respectively), terms related to the ribosome are not significantly enriched (p-values  > 0.3). On the other hand, terms related to “amino acid metabolic process” remain significant both for flies and humans, using either nucleotide or amino acid similarities. Other significant GO terms appear that are not very specific, for example “nucleotide binding” or “mitochondrion” for human genes when using amino acid similarity. On the one hand, these results suggest that the significant enrichment of ribosome-related functions is restricted to genes highly conserved between flies and humans and yet highly variable within these species. On the other hand, we note that these results may also indicate that the enrichment of the term “amino acid metabolic process” could be a possible artifact caused by the procedure by which genes are selected, since the effect still appears at the less restrictive cut-off.

Nevertheless, the fact that we observe both for humans and flies the same functions for genes that are highly conserved between species and highly variable within species, namely mRNA translation, might reflect positive selection for variation in proteins that are targeted by viruses. We hypothesize the existence of mechanisms to increase the mutation rates in regions of these proteins that to do not negatively affect their function but allow escape from viral control. Variability within species confers a protection from viruses since this prohibits them from targeting all members of a species.

We did not observe great differences in functional analysis whether studying nucleotide or amino acid variations, suggesting that the observed conservation affects the translated proteins.

We observed, however, some differences between humans and flies, namely gene functions related to amino acid catabolism and tRNA synthesis in selected fly genes. Regarding these differences, we note that in a study comparing experimental results on the genetic architectures of quantitative loci of flies, mice and humans, a remarkable amount of agreement was noted between species, with differences attributed to experimental design [17]. In this respect, one very significant difference between the sequencing projects compared here is the characteristics of the cohorts sequenced. The 39 fruit fly strains analyzed in [2] were taken from the DGRP, which consists of 192 inbred strains derived from a single outbred population (Raleigh, USA population [18]). In contrast, the human genomes originated from several geographically distant populations (Europe, East Asia, sub-Saharan Africa and the Americas [1]). Accordingly, we observed that the protein G6PD (approximately 62% conserved between humans and flies), whose malaria resistance-deficient version is observed in regions where this disease is endemic [19], ranked very high in variability in humans (in nucleotides or amino acids, rank #23 and #31, respectively) but not in *Drosophila* (orthologous gene FBgn0004057, a.k.a. Zw, in nucleotides or amino acids, rank #1539 and #2261, respectively). The different properties of the fly and human cohorts underlying our analysis have to be taken into account when examining our results.

In conclusion, our analysis may have uncovered a list of genes in fly and humans that display traces of positive selection for intraspecies variation among a background of genes with highly conserved sequences and likely essential functions. The detection of functions related to mRNA translation might open a view of a dramatic battle between eukaryotes and viruses that has been going on for several eons. But other functions hint towards undiscovered processes that increase gene variability in fly and human populations. Our analysis will facilitate the study of such processes and might be expanded as extended genome populations from humans, flies, and other species are sequenced.

## Methods

### Relevant orthologs

Confirmed orthologs between *D. melanogaster* and *H. sapiens* were obtained from Ensembl Genes Release 72 (www.ensembl.org/biomart/martview), with a total of 9697 human genes and 7386 fly genes. Several filters were applied to fit the following criteria: 1) both genes were longer than 300 base pairs; 2) the shorter gene (length measured in base pairs) no shorter than 70% of its longer ortholog; and 3) only one-to-one ortholog pairs after filters 1 and 2. This filter resulted in 3092 fly-to-human ortholog pairs.

### Percentage identity

Amino acid percentage identities of orthologs between fruit flies and humans were obtained from Ensembl Genes Release 72 (www.ensembl.org/biomart/martview). Nucleotide percentage identities were obtained by downloading aligned nucleotide sequences from Ensembl's REST API (Release 72) and determining similarity between orthologs.

### Variant data

Variant calls of *D. melanogaster* were obtained from data derived from the *Drosophila* Genetic Reference Panel (DGRP) [2] (http://dgrp.epfl.ch/downloads/). These contain information on single-nucleotide, multi-nucleotide, and structural variants in 39 inbred lines, in chromosomes 2 L, 2R, 3 L, 3R, 4, and X. Variant calls of *H. sapiens* were obtained from the 1000 Genomes project [1] phase 1 release v3 at the EBI ftp server (ftp://ftp.1000genomes.ebi.ac.uk/vol1/ftp/release/20110521/). These contain single-nucleotide, multi-nucleotide, and structural variants from 1092 samples from four subpopulations across the globe, in chromosomes 1–22 and X. The 10 ortholog pairs contained in human mitochondrial DNA, which has no variance data, were excluded, yielding 3082 relevant ortholog pairs.

Variant calls were filtered to contain only single-nucleotide polymorphisms (SNPs) in coding sites of the 3082 orthologous human and 3082 orthologous fly genes. Raw allele counts were then computed using the VCFtools (v0.1.11) software [20] — counts option. For each gene, all allele counts except for the highest frequency alleles at each SNP were summed and divided by the length (in base pairs) of the gene coding region to arrive at the nucleotide variance. For amino acid variance, SNP sites were first run through the Ensembl API's Variant Effect Predictor (VEP) script to obtain information about functional consequences of each variant and filtered to contain non-synonymous variants exclusively. Only the allele counts of these specific SNPs were summed and divided by the length (in base pairs) of the gene coding region to arrive at the amino acid variance.

The following are the supplementary data related to this article.

Supplementary Table 1 Scored pairs of orthologous fly and human genes.

Supplementary Table 2 Selected gene ontology terms enriched in genes with high conservation between species and high intra-species variability.

## Figures and Tables

**Table 1:** Enriched gene ontology terms. For each cell in the table: (i) number of genes tested (annotated with any GO terms), (ii) definition of the enriched GO term, (iii) identifier, (iv) symbols of the genes with the GO term found in the test set, (v) number of genes found of all annotated with the GO term, and (vi) p-value indicating the significance of the enrichment. A larger list is shown as Supplementary Table 2.

	nt conservation	aa conservation
Fly	94 genes	34 genes
Structural constituent of ribosome	Amino acid metabolic process
GO: 0003735	GO: 0006520
mrps7 tko rps23 rpl27a rpl13 cg4866 rpl23 rps26 rpl8 rpl27 mrpl14 rpl7a rps15 rps9	cg9547 aats-gly aats-asn aats-arg cg10361 aats-gln aats-glupro cg6415
14 of 111	8 of 52
0.0312	0.00073
Human	28 genes	24 genes
Cytosolic small ribosomal subunit	Cytosolic ribosome
GO: 0005843	GO: 0005830
rps16 rps25 rps9	rps2 rplp0 rps16 rps23 rps9 rpl8
3 of 17	6 of 43
0.13	0.000723
